# An approximation theorem and generic convergence for equilibrium problems

**DOI:** 10.1186/s13660-018-1617-y

**Published:** 2018-02-05

**Authors:** Xiaoling Qiu, Wensheng Jia, Dingtao Peng

**Affiliations:** 1 School of Mathematics and Statistic, Guizhou University, Guiyang, China; 2 College of Science and Technology, Guizhou University, Guiyang, China

**Keywords:** Equilibrium problems, Approximation theorem, Generic convergence, Set-valued mapping, Pseudocontinuity

## Abstract

In this paper, we prove an approximation theorem for equilibrium problems and provide theoretical support for many algorithms. Simon’s bounded rationality is illustrated by an approximation theorem, that is, bounded rationality is approaching full rationality as its ultimate goal. Furthermore, by the methods of set-valued analysis, we obtain the generic uniqueness and generic convergence of the solutions of monotone equilibrium problems in the sense of Baire category. As applications, we investigate the optimization problem, variational inequality problem and saddle point problem as special cases.

## Introduction

Equilibrium problems (in short, EP) by Blum and Oettli [1] provide a mathematical framework which includes optimization, variational inequalities, fixed point and saddle point problems and noncooperative games as particular cases. Actually, the Ky Fan minimax inequality [2] is considered to be earlier form of the equilibrium problems. The general format is as the following:

$$\mbox{find }x^{*}\in C\mbox{ such that }f\bigl(x^{*},y\bigr)\geq0\quad \mbox{for all }y \in C, $$

where *C* is a nonempty closed subset of a metric space *X* and $f:X\times X\to R$ is a scalar function. The set *C* is called a constraint set and the function *f* is called a bifunction or objective function.

This general format has received more and more interest in the last decade mainly because many theoretical and algorithmic results developed through the unifying language. The research on equilibrium problems itself basically has the following three aspects: the first is about the existence of the solutions of equilibrium problems, and there are fruitful results achieved in [3–6] and the references therein; the second is to study the stability of the equilibrium point of equilibrium problems, including the well-posedness and generic stability, and many works involved in [7–11]; the third aspect is, in recent years, the solution of the approximation as the hot topic for many scholars, which involves in the promotion problems associated with the equilibrium problems, such as quasiequilibrium problems, quasivariational problem, approximation of vector equilibrium problems, etc.; see [8, 12–17] and the literature therein. A lot of different algorithms have been designed to implement the approximate solution of specific problems. It is natural to ask if one can make these all kinds of algorithms to a unification of the theory? How does it depend on the objective function, constraint set and the solving method? This will be answered in this paper.

On the other hand, according to Simon’s bounded rationality theory [18], there are three factors to influence the outcome of decisions. They are as follows: each decision maker can never fully understand all the alternatives in front of himself and may choose approximate strategies; each decision maker chooses approximate functions as objective functions; the solving methods are also approximate during the specific calculation process. In a word, there are three approximate ways. Therefore, the decision maker is to seek “satisfactory solution” rather than “optimal solution”. From the point of view of practical significance, it reflects that bounded rationality is approaching full rationality as its ultimate goal.

In this paper, our aim is to construct an approximation theorem of equilibrium problems and provide theoretical support for a number of algorithms on the equilibrium problems. The Fort theorem plays a critical role in generic uniqueness and convergence of approximate solutions under some assumptions of mild continuity. We investigate the applications of such approximation theorem in optimization problem, variational inequality problem and saddle point problem as our special cases.

This paper is organized as follows. In Section 2, we recall some definitions and necessary lemmas which shall be used in the sequel. We prove an important approximation theorem and give the detailed remarks in Section 3. In Section 4, we analyze the uniqueness of the solution and make use of the Fort theorem to obtain the generic convergence on monotonous equilibrium problems. Some applications are given to investigate our theoretical results in Section 5. Section 6 concludes this paper.

## Preliminaries

Now let us recall some definitions and lemmas which we will use.

### Definition 2.1

([19, 20])

Let *X* be a Hausdorff topological space and $f:X\to R$ be a function.

1. *f* is said to be upper pseudocontinuous at $x_{0}\in X$ if for all $x\in X$ such that $f(x_{0})< f(x)$, we have
  $$\limsup_{y\rightarrow x_{0}}f(y)< f(x); $$
  *f* is said to be upper pseudocontinuous on *X* if it is upper pseudocontinuous at each *x* of *X*;
2. *f* is said to be lower pseudocontinuous at $x_{0}\in X$ if −*f* is upper pseudocontinuous at $x_{0}$; *f* is said to be lower pseudocontinuous on *X* if it is lower pseudocontinuous at each *x* of *X*;
3. *f* is said to be pseudocontinuous at $x\in X$ if *f* is both upper pseudocontinuous and lower pseudocontinuous at *x*; *f* is said to be pseudocontinuous on *X* if *f* is pseudocontinuous at each *x* of *X*.

### Remark 2.1

Each upper (resp. lower) semicontinuous function is also upper (resp. lower) pseudocontinuous. But the converse is not true; see the following counterexample.

Let $X=[0,2]$, $f_{i}: X\to R$, $i=1,2$,

$$\begin{aligned} f_{1}(x):= \textstyle\begin{cases} -x, & 0\leq x< 1, \\ -2, & 1\leq x< 2. \end{cases}\displaystyle ;\qquad f_{2}(x):= \textstyle\begin{cases} x-1,& 0\leq x< 1, \\ 1,& 1\leq x< 2. \end{cases}\displaystyle \end{aligned}$$

We can easily check that $f_{1}$ is upper pseudocontinuous but not upper semicontinuous at $x=1$ and that $f_{2}$ is not lower semicontinuous but lower pseudocontinuous at $x=1$.

We need to use the methods of set-valued analysis in our paper to investigate the generic uniqueness and convergence. The following definitions and lemmas are necessary; for more details, see [21].

### Definition 2.2

Let *X*, *Y* be two metric spaces. Denote by $P_{0}(Y)$ the space of all nonempty subsets of *Y*. $F: X\to P_{0}(Y)$ is a set-valued mapping. Then (1) *F* is said to be upper (lower, respectively) semicontinuous at $x\in X$ if for each open set *G* in *Y* with $G\supset F(x)$ ($G\cap F(x)\neq\emptyset$, respectively), there exists an open neighborhood *O* of *x* such that $G\supset F(x')$ ($G\cap F(x')\neq \emptyset$, respectively) for each $x'\in O$; (2) *F* is said to be continuous at $x\in X$ if *F* is both upper semicontinuous and lower semicontinuous at *x*; and *F* is said to be continuous on *X* if *F* is continuous at each $x\in X$; (3) *F* is said to be closed if the graph of *F* $\operatorname{Graph}(F)= \{(x,y)\in X\times Y: y\in F(x)\}$ is closed.

### Remark 2.2

If $F: X\to P_{0}(Y)$ is closed and *Y* is compact, then *F* is upper semicontinuous at each $x\in X$.

### Remark 2.3

In [21], examples show the difference between the upper semicontinuity and lower semicontinuity. None of them could include the other one, but the following Fort theorem [22] refers to the connection between them.

### Fort theorem

*Let* *X* *be a Hausdorff topological space*, *Y* *be a metric space and* $F: X\to P_{0}(Y)$ *be an upper semicontinuous compact mapping*. *Then there exists a residual subset* *Q* *of* *X* *such that*, *for all* $x\in Q, F$ *is lower semicontinuous at* $x\in Q$.

### Remark 2.4

If *X* is a Baire space, then the residual subset of *X* must be dense in *X*. Based on this, the Fort theorem has another form as Lemma 2.3 of [23] by Tan, Yu and Yuan. Moreover, the Fort theorem has been improved to adapt non-compactness cases by Xiang, etc (see[24]).

### Remark 2.5

If there exists a dense residual subset *Q* of *X* such that $\forall x\in Q$, certain property *P* depending on *x* holds, then we say the property *P* is generic on *X*. At this point, the reader should be referred to the recent book by Reich and Zaslavski (see [25]).

The following three lemmas play a critical role for our approximation theorem, which can be seen as Lemma 2.1.4, Theorem 2.1.2 and Lemma 2.1.6 in [26]. For the completeness of the structure of the paper, we write down the proofs. Let $(X,d)$ be a metric space and *A*, *B* be nonempty bounded subsets of *X*, a real number $\sigma>0$, denoted by

$$U(\sigma,A)=\bigl\{ x\in X:\mbox{ there exists }a\in A\mbox{ such that }d(x,a)< \sigma\bigr\} . $$

It is obvious that $U(\sigma,A)$ is an open subset of *X*. The Hausdorff distance[21] on *X* between the sets *A* and *B* is defined as

$$h(A,B)=\inf\bigl\{ \sigma>0: A\subset U(\sigma, B)\mbox{ and }B\subset U(\sigma, A)\bigr\} . $$

### Lemma 1

*Let* $(X,d)$ *be a metric space and* *G* *be an open subset of* *X*, *and let* *A* *be a nonempty compact subset of* *X*. *If* $G\supset A$, *then there exists a real number* $\sigma>0$ *such that* $G\supset U(\sigma,A)$.

### Proof

Assume that, by contradiction, there exists $G\not\supset U(\sigma_{n},A)$ for any $\sigma_{n}>0$, $\sigma_{n}\to0$ (*n* is an integer and $n\to\infty$). Then there exists a sequence $\{x_{n}\}_{n=1}^{\infty }\subset U(\sigma_{n},A)$, but $x_{n}\notin G$. By the definition of $U(\sigma_{n},A)$, there is a sequence $\{y_{n}\}_{n=1}^{\infty}\subset A$ such that $d(x_{n},y_{n})\leq\sigma_{n}$ for any $n=1,2,\ldots$ . By the compactness of *A*, there exists a subsequence $\{y_{n_{k}}\}$ of $\{y_{n}\} $ converging to $y^{*}$ and $y^{*}\in A$. Without loss of generality, set $y_{n}\to y^{*}$, $n\to\infty$. By the metric triangle inequality,

$$d\bigl(x_{n},y^{*}\bigr)\leq d(x_{n},y_{n})+d \bigl(y_{n},y^{*}\bigr)\to0 \quad(n\to\infty), $$

we have $x_{n}\to y^{*}$. $x_{n}\notin G$ implies $y^{*}\notin G$. In fact, $y^{*}\in A\subset G$, that is a contradiction. □

### Lemma 2

*Assume that for* $n=1,2,\ldots$ , *there is a nonempty bounded subset sequence* $A_{n}$ *of* *X* *and* *A* *is a nonempty compact subset of* *X*. *If* $x_{n}\in A_{n}$ *and* $h(A_{n},A)\to0$, *where* $h(A_{n},A)$ *means the Hausdorff distance on* *X*, *then there exists a subsequence* $\{x_{n_{k}}\}$ *of* $\{ x_{n}\}$ *such that* $x_{n_{k}}\to x^{*}$ ($n\to\infty$) *and* $x^{*}\in A$.

### Proof

By the way of contradiction, if the result is not true, then for any point $x\in A$, there exist a neighborhood $O(x)$ of *x* and a positive integer $N(x)$ such that for any $n\geq N(x)$, $x_{n}\notin O(x)$. We have $\bigcup_{x\in A}O(x)\supset A$. Since *A* is compact, there exist finite points denoted by $x^{1},x^{2},\ldots,x^{k}\in A$ such that $\bigcup_{i=1}^{k}O(x^{i})\supset A$. Denote by $G=\bigcup_{i=1}^{k}O(x^{i})$, hence $G\supset A$ and *G* is an open set. By Lemma 1, there exists $\sigma>0$ such that $G\supset U(\sigma,A)$. □

Note that $h(A_{n},A)\to0$ ($n\to\infty$), there exists a positive integer *N* such that for any $n\geq N$, $h(A_{n},A)<\sigma$. Consequently, $A_{n}\subset U(\sigma,A)\subset G$. Setting $N_{0}\geq\max\{ N(x^{1}),N(x^{2}),\ldots, N(x^{k})\}$, then for any $n\geq N_{0}$, $x^{n}\notin O(x^{i})$, $i=1,2,\ldots,k$. Therefore $x_{n}\notin G$, which is a contradiction to $x_{n}\in A_{n}\subset G$.

### Lemma 3

*For* $n=1,2,\ldots$ , *let* $A_{n}$ *be a nonempty bounded subset of* *X* *and* *A* *be a nonempty subset of* *X*. *Let* *G* *be an open set of* *X*. *If* $G\cap A\neq\emptyset$ *and* $h(A_{n},A)\to0$, *then there exists a positive integer* *N* *such that*, *for any* $n\geq N$, $G\cap A_{n}\neq \emptyset$.

### Proof

Since $G\cap A\neq\emptyset$, we take some point $x\in G\cap A$, then $x\in G$ and $x\in A$. There exists $\sigma>0$ such that $O(x,\sigma)\subset G$ by the openness of *G*. Note that $h(A_{n},A)\to 0$, then there exists a positive integer *N* such that, for any $n\geq N$, $h(A_{n},A)<\sigma$. Hence we have $A\subset U(\sigma, A_{n})$. Since $x\in A$, there exists $x_{n}\in A_{n}$ such that $x_{n}\in O(x,\sigma )\subset G$. Then $G\cap A_{n}\neq\emptyset$. □

## An approximation theorem

In this section, we are interested in the approximation solutions of (EP) associated with both the constraint set and the objective function of (EP). To this purpose, we introduce a definition of approximate equilibrium point. Now we start our analysis with (EP).

### Definition 3.1

Let *X* be a metric space, and given a function $f:X\times X\to R$ and a real number $\epsilon>0$, $x^{*}$ is said to be an *ϵ*-equilibrium point of *f* if

$$f\bigl(x^{*},y\bigr)\geq-\epsilon,\quad \forall y\in X. $$

The *ϵ*-equilibrium point is strict if the above inequality is strict for all $y\neq x^{*}$.

### Theorem A

*Let* *X* *be a metric space and all the following assumptions be satisfied*:

1. *For any* $n=1,2,\ldots$ , *the function sequence* $f^{n}:X\times X\to R$ *is satisfied with* $\sup_{(x,y)\in X\times X}| f^{n}(x,y)-f(x,y)| \to0$, *where the function* $f:X\times X\to R$ *is upper pseudocontinuous*;
2. *For any* $n=1,2,\ldots$ , $A_{n}$ *is a subset sequence of* *X* *with* $h(A_{n},A)\to0$ ($n\to\infty$), *where* *A* *is a nonempty compact subset of* *X* *and* $f(x,x)=0$ *for any* $x\in A$;
3. *For any* $n=1,2,\ldots$ , $x_{n}\in A_{n}$ *is an* $\epsilon _{n}$-*equilibrium point of* $f^{n}$, *that is*, $f^{n}(x_{n},y)\geq-\epsilon_{n}$ *for any* $y\in A_{n}$, *where* $\epsilon_{n} >0$ *and* $\epsilon_{n}\to0$ ($n\to \infty$).

*Then*

1. *there exists a convergent subsequence* $\{x_{n_{k}}\}$ *of the sequence* $\{x_{n}\}$ *and the limit belongs to* *A*, *that is*, $x_{n_{k}}\to x^{*}$ ($n\to\infty$) *and* $x^{*}\in A$;
2. *for any* $y\in A$, $f(x^{*},y)\geq0$.

### Proof

(1) It is obvious from Lemma 2 and the proof can be omitted.

(2) It follows from (1) that we may assume that $x_{n}\to x^{*}$. If it is not true, then there exists some point $y_{0}\in A$ such that $f(x^{*},y_{0})<0$. Since $f(x^{*},x^{*})=0$, then $f(x^{*},y_{0})< f(x^{*},x^{*})$. By the definition of upper pseudocontinuity of *f* at $(x^{*},y_{0})$, we have

$$\limsup_{x'\to x^{*},y'\to y_{0}}f\bigl(x',y'\bigr)< f \bigl(x^{*},x^{*}\bigr)=0. $$

Hence there exists $\sigma_{0}>0$ such that

$$\limsup_{x'\to x^{*},y'\to y_{0}}f\bigl(x',y'\bigr)< - \sigma_{0}. $$

From Proposition 2.1 of [20], there exist a neighborhood $O(x^{*})$ of $x^{*}$ and a neighborhood $O(y_{0})$ of $y_{0}$ such that $f(x',y')<-\sigma_{0}$ for any $x'\in O(x^{*})$ and any $y'\in O(y_{o})$.

Note the conditions that $\sup_{(x,y)\in X\times X}|f^{n}(x,y)-f(x,y)|\to0$ and $\epsilon_{n}\to0$ ($n\to\infty$), there exists a positive integer $N_{1}$ such that, for any $n\geq N_{1}$,

$$\sup_{(x,y)\in X\times X} \bigl\vert f^{n}(x,y)-f(x,y) \bigr\vert < \frac{\sigma_{0}}{2} \quad \mbox{and}\quad \epsilon_{n}< \frac{\sigma_{0}}{2}. $$

Since $n\to\infty$, $x_{n}\to x^{*}$, $h(A_{n},A)\to0$ and $y_{0}\in A$, by Lemma 3, there exists a positive integer $N_{2}\geq N_{1}$ such that, for any $n\geq N_{2}$, $x_{n}\in O(x^{*})$ and $O(y_{0})\cap A_{n}\neq\emptyset $. We take $y_{n}\in O(y_{0})\cap A_{n}$. Hence we have

$$f(x_{n},y_{n})< -\sigma_{0} $$

and

$$f^{n}(x_{n},y_{n})< f(x_{n},y_{n})+ \frac{\sigma_{0}}{2}< -\frac{\sigma_{0}}{2}< -\epsilon_{n}. $$

That is a contradiction since $x_{n}$ is an $\epsilon_{n}$-equilibrium point of $f^{n}$. Thus this completes the proof. □

In particular, in Theorem A if $A_{n}=A$, $n=1,2,\ldots$ , and the assumption of $f(x,y)$ can be weakened to *f* is upper pseudocontinuous in the first variable on *X* for any $y\in A$, then the result holds, see Corollary 3.1.

### Corollary 3.1

*Let* *X* *be a metric space and all the following assumptions be satisfied*:

1. *For any* $n=1,2,\ldots$ , *the function sequence* $f^{n}:X\times X\to R$ *is satisfied with* $\sup_{(x,y)\in X\times X}| f^{n}(x,y)-f(x,y)| \to0$, *where the function* $f:X\times X\to R$ *is upper pseudocontinuous in the first variable*;
2. *A* *is a nonempty compact subset of* *X* *and* $f(x,x)=0$ *for any* $x\in A$;
3. *For any* $n=1,2,\ldots$ , $x_{n}\in A$ *is an* $\epsilon _{n}$-*equilibrium point of* $f^{n}$, *that is*, $f^{n}(x_{n},y)\geq-\epsilon_{n}$ *for any* $y\in A$, *where* $\epsilon_{n} >0$ *and* $\epsilon_{n}\to0$ ($n\to \infty$).

*Then*

1. *there exists a convergent subsequence* $\{x_{n_{k}}\}$ *of the sequence* $\{x_{n}\}$ *and the limitation belongs to* *A*, *that is*, $x_{n_{k}}\to x^{*}$ ($n\to\infty$) *and* $x^{*}\in A$;
2. *for any* $y\in A$, $f(x^{*},y)\geq0$.

### Remark 3.1

From Remark 2.1, Theorem A retains its validity when $f:X\times X\to R$ is continuous or upper semicontinuous on $X\times X$.

### Remark 3.2

Theorem A is meaningful and widely used in all kinds of algorithms of (EP). The significance is that an approximate sequence $\{ x_{n}\}$ is obtained despite the approximate functions (*f* replaced by the approximate functions $f^{n}$), the approximate feasible set ($A_{n}$ instead of *A*) and the approximate calculation method ($\epsilon _{n}$-equilibrium point). What is more important, there exists a convergent subsequence $\{x_{n_{k}}\} $ of $\{x_{n}\}$ and the convergence point is the solutions of objective function *f*, that is, $f(x,y)\geq 0$ for any $y\in A$. If we see the solutions of *f* as the optimal solutions under full rationality and $\epsilon_{n}$-equilibrium of $f^{n}$ as the approximate solutions under bounded rationality, Theorem A reflects that the bounded rationality is approximate to full rationality (see Simon [18]).

### Remark 3.3

In fact Theorem A implies that every sequence $\{x_{n}\}$ must have a convergent subsequence and every convergent point belongs to the set *A*. If the function $f(x,y)$ has a unique solution $x^{*}$ on *A*, then there must be $x_{n}\to x^{*}$.

### Remark 3.4

We cannot ensure that $f^{n}(x,y)$ must be pseudocontinuous on $X\times X$ for $n=1,2,\ldots$ , even if $f(x,y)$ is pseudocontinuous on $X\times X$ and $\sup_{(x,y)\in X\times X}|f^{n}(x,y)-f(x,y)|\to0$ ($n\to\infty$).

### Remark 3.5

We cannot guarantee that the set sequence $A_{n}$ must be compact on *X*, while *A* is a nonempty compact set on *X* and $h(A_{n},A)\to0$ ($n\to \infty$). There is a simple example that $X=R$, $A=[0,1]$, $A_{n}=(\frac {1}{n},1-\frac{1}{n})$. It is easy to see that $h(A_{n},A)\to0$ ($n\to \infty$) but $A_{n}$ is open.

## Generic convergence

In this section, we transfer our interest to the uniqueness and the convergence solution of (EP). Firstly we construct an equilibrium problems’ space under some assumptions and study the continuity of the set of solutions under the uniform metric on the objective functions. We will show that the set-valued mapping on the solutions of (EP) is upper semicontinuous and compact on this space. Then we consider a subspace of this space in which the equilibrium problems have a monotonicity property.

Assume that $(X,d)$ is a nonempty compact metric space.

The problem space *C* is given by

$$C=\left \{ f:X\times X\rightarrow R: \textstyle\begin{array}{l} \forall x\in X, f(x,x)=0;\\ \forall y\in X, x\to f(x,y)\mbox{ is upper semicontinous on }X;\\ \forall x,y\in X, \displaystyle\sup_{(x,y)\in X\times X} \vert f(x,y) \vert < +\infty;\\ \mbox{there exists }x\in X\mbox{ such that }f(x,y)\geq0\mbox{ for any }y\in X. \end{array}\displaystyle \right \} $$

For any $f_{1},f_{2}\in C$, define

$$\rho(f_{1},f_{2})=\sup_{(x,y)\in X\times X} \bigl\vert f_{1}(x,y)-f(x,y) \bigr\vert . $$

It is easy to check that $(C,\rho)$ is a complete space.

For any $f\in C$, denote by $F(f)$ all the solutions of *f*, i.e., $F(f)=\{x\in X: \forall y\in X, f(x,y)\geq0\}$. By the definition of *C*, we know $F(f)\neq\emptyset$. Then the correspondence $f\to F(f)$ defines a set-valued mapping $F: C\to P_{0}(X)$, and the following lemma shows the compactness and semicontinuity of the set-valued mapping.

### Lemma 4.1

*The set*-*valued mapping* $F: C\to P_{0}(X)$ *is compact and upper semicontinuous on* *C*.

### Proof

Since *X* is compact, by Remark 2.2, it suffices to show that the graph of *F* is closed, where $\operatorname{Graph}(F)=\{(f,x)\in C\times X: x\in F(f)\}$. Suppose that $f_{n}\in C$ with $f_{n}\to f$ and $x_{n}\in F(f_{n})$ with $x_{n}\to x$, we shall show $x\in F(f)$.

For any $y\in X$, $\forall n=1,2,\ldots$ , it follows from $x_{n}\in F(f_{n})$ that $f_{n}(x_{n},y)\geq0$. Since $f_{n}\to f$, $x_{n}\to x$ and *f* is upper semicontinuous at *x*, we have

$$\begin{aligned} f(x,y)&\geq\limsup_{n}f(x_{n},y) \\ &=\limsup_{n}\bigl[\bigl(f(x_{n},y)-f_{n}(x_{n},y) \bigr)+f_{n}(x_{n},y)\bigr] \\ &\geq\limsup_{n}\bigl[f(x_{n},y)-f_{n}(x_{n},y) \bigr] \\ &\geq\lim_{n}(-\rho(f_{n},f)=0. \end{aligned} $$

Therefore $x\in F(f)$ and this completes the proof. □

### Remark 4.1

By the Fort theorem and Lemma 4.1, the generic continuity holds, that is, there exists a dense residual subset *Q* of *C* such that $\forall f\in Q$, *F* is continuous at *f*.

In the following, we consider the subset $C_{1}$ of *C* where the function *f* has a monotonicity property for the generic uniqueness.

Denote by

$$C_{1}=\left \{f\in C: \textstyle\begin{array}{l} f \mbox{ is monotone on } X\times X,\\ \mbox{i.e.}, \forall x,y\in X, f(x,y)+f(y,x)\leq0. \end{array}\displaystyle \right \} $$

It is easy to see that $C_{1}$ is closed in *C* and $(C_{1},\rho)$ is also a complete space.

### Theorem 4.1

*There exists a dense residual subset* $Q_{1}$ *of* $C_{1}$ *such that* $\forall f\in Q_{1}$, $F(f)$ *is a singleton set*.

### Proof

By the Fort theorem, there exists a dense residual subset $Q_{1}$ of $C_{1}$ such that, for any $f\in Q_{1}$, the set-valued mapping *F* is lower semicontinuous at *f*.

Assume, by contradiction, that $F(f)$ is not a singleton set for some $f\in Q_{1}$. Then there exist at least two points $x_{1},x_{2}\in F(f)$ and $x_{1}\neq x_{2}$. And there exist an open neighborhood *V* of $x_{1}$ and an open neighborhood *W* of $x_{2}$ such that $V\cap W= \emptyset$.

$\forall x\in X$, define the function $h:X\to[0,1]$ as follows:

$$h(x)=\frac{d(x,x_{1})}{d(x,x_{1})+d(x,X\backslash V)}, $$

where $d(x,X\backslash V)=\inf_{y\in X\backslash V}d(x,y)$.

It is obvious that $h(x)$ is continuous at each $x\in X$ and $h(x_{1})=0$, $h(x)=1$ for any $x\in X\backslash V$. Specially, there must be $h(x)=1$ for any $x\in W$ since $W\subset X\backslash V$ and hence $h(x_{2})=1$.

$\forall x\in X$, $\forall y\in X$, $\forall n=1,2,\ldots$ , define the function $f_{n}:X\times X\to R$ as follows:

$$f_{n}(x,y)=f(x,y)+\frac{1}{n}\bigl[h(y)-h(x)\bigr]. $$

It is easily checked that $f_{n}\in C_{1}$ and $\rho(f_{n},f)\to0$ ($n\to \infty$).

Note that $x_{2}\in F(f)\cap W$, then $F(f)\cap W\neq\emptyset$. Since *F* is lower semicontinuous at *f* and $f_{n}\to f$, there exists a positive integer $n_{0}$ large enough such that $F(f_{n_{0}})\cap W\neq \emptyset$. Take $x_{n_{0}}\in F(f_{n_{0}})\cap W$, then $x_{n_{0}}\in W$ and $x_{n_{0}}\in F(f_{n_{0}})$. We have $h(x_{n_{0}})=1$ and $f_{n_{0}}(x_{n_{0}},y)\geq0$ for any $y\in X$. Especially, $f_{n_{0}}(x_{n_{0}},x_{1})\geq0$. Since $f\in C_{1}$ and $x_{1}\in F(f)$, we have $f(x_{n_{0}},x_{1})\leq-f(x_{1},x_{n_{0}})\leq0$. On the other hand, from the definition of $f_{n_{0}}$ we get

$$f_{n_{0}}(x_{n_{0}},x_{1})=f(x_{n_{0}},x_{1})+ \frac {1}{n_{0}}\bigl[h(x_{1})-h(x_{n_{0}})\bigr]\leq- \frac{1}{n_{0}}< 0. $$

That is a contradiction to $f_{n_{0}}(x_{n_{0}},y)\geq0$, $\forall y\in X$. Therefore $F(f)$ must be a singleton set. □

### Remark 4.2

$\forall f\in C_{1}$, *f* is pseudocontinuous when *f* is monotone on $X\times X$. Since $\sup_{(x,y)\in X\times X}|f_{n}(x,y)-f(x,y)|\to0$ ($n\to\infty$) and $f_{n}(x_{n},y)\geq-\epsilon_{n}$ for any $y\in X$, where $\epsilon_{n}>0$, $\epsilon_{n}\to0$, there is a sequence $\{x_{n}\}\subset X$. All the conditions of Theorem A are satisfied and Theorem A holds.

Combining Theorem A and Theorem 4.1, we can obtain the generic convergence of solutions of (EP).

### Theorem 4.2

*Let* $x_{n}$ *be an* $\epsilon_{n}$-*equilibrium point of* $f_{n}$ *with the convergence function* *f* *under the uniform metric*. *Then there exists a dense residual subset* $Q_{2}$ *of* $C_{1}$ *such that*, *for any* $f\in Q_{2}$, *f* *has a unique solution* $x^{*}$ *with* $x_{n}\to x^{*}$ *and* $f(x^{*},y)\geq0$ *for all* $y\in X$.

## Applications

In [1, 17] it is shown that several problems in nonlinear analysis can be written as (EP). In this section we deal with various such problems as applications of Theorem A.

### Minimization problem

Let $h: D\subseteq X\to R$ be a function with *D* being a closed set. The minimization problem is defined as

$$\mbox{find }\overline{x}\in D \mbox{ such that } h(y)\geq h(\overline {x}),\quad \forall y\in D. $$

Setting $f(x,y)=h(y)-h(x)$, we have that the minimization problem is equivalent to (EP).

By Theorem A, we give the following Proposition 5.1.

#### Proposition 5.1

*Assume that the following assumptions are satisfied*:

1. *For each* $n=1,2,\ldots$ , $h^{n}: D\to R$ *is the function sequence with* $\sup_{x\in D}|h^{n}(x)-h(x)|\to0$ ($n\to\infty$), *where* $h: D\to R$ *is pseudocontinuous*;
2. *For each* $n=1,2,\ldots$ , $A_{n}$ *is a nonempty subset of* *D* *with* $h(A_{n},A)\to0$ ($n\to\infty$), *where* *A* *is a nonempty subset of* *D*;
3. *For each* $n=1,2,\ldots$ , $x_{n}\in A_{n}$ *satisfies*
  $$h^{n}(x_{n})\leq\inf_{u\in A_{n}}h^{n}(u)+ \epsilon_{n}, $$
  *where* $\epsilon_{n}\geq0$, $\epsilon_{n}\to0 $ ($n\to\infty$).

*Then*

1. *there must exist a convergent subsequence* $\{x_{n_{k}}\}$ *of the sequence* $\{x_{n}\}$ *such that* $x_{n_{k}}\to x^{*}$ ($n\to\infty$) *and* $x^{*}\in A$.
2. $h(x^{*})=\min_{u\in A}h(u)$.

#### Proof

$\forall x,y\in D$, let $f(x,y)=h(y)-h(x)$ and $f^{n}(x,y)=h^{n}(y)-h^{n}(x)$ for any $n=1,2,\ldots$ . Then it is easy to check that all the conditions of Theorem A are satisfied and it is natural to obtain the results (1) and (2). This completes the proof. □

### Variational inequality problem

Let $T: K\subseteq R^{n}\to R^{n}$ be a function with *K* being a closed set, where $R^{n}$ means an *n*-dimensional Euclidean space. The variational inequality problem is defined as follows:

$$\mbox{find }\overline{x}\in K \mbox{ such that } \bigl\langle T(\overline {x}),y-\overline{x}\bigr\rangle \geq0, \quad\forall y\in K, $$

where $\langle\cdot\rangle$ means the product of $R^{n}$.

Setting $f(x,y)=\langle T(x),y-x\rangle$ for any $x,y\in K$, we have that the problem is equivalent to (EP).

For $n=1,2,\ldots$ , let $T^{n}: K\subseteq R^{n}\to R^{n}$ be a function sequence approximate to *T* with the uniform metric, where $T: K\subseteq R^{n}\to R^{n}$ is continuous. And then we define $f^{n}(x,y)=\langle T^{n}(x),y-x\rangle$. According to Theorem A, we obtain the following Proposition 5.2.

#### Proposition 5.2

*Let* *T*, *f*, $\{f^{n}\}$, *K*, $\{K_{n}\}$ *be as in the above*. *If the following assumptions are satisfied*:

1. *For each* $n=1,2,\ldots$ , $T^{n}: K\to R^{n}$ *is the function sequence with* $\sup_{x\in K}|T^{n}(x)-T(x)|\to0$ ($n\to\infty$), *where* $T: K\subseteq R^{n}\to R^{n}$ *is continuous*;
2. *For each* $n=1,2,\ldots$ , $K_{n}$ *is a nonempty subset of* $R^{n}$ *with* $h(K_{n},K)\to0$ ($n\to\infty$), *where* *K* *is a nonempty subset of* $R^{n}$;
3. *For each* $n=1,2,\ldots$ , $x_{n}\in K_{n}$ *satisfies*
  $$\bigl\langle T^{n}(x_{n}),y-x_{n}\bigr\rangle \geq- \epsilon_{n},\quad \forall y\in K_{n}, $$
  *where* $\epsilon_{n}\geq0$, $\epsilon_{n}\to0$ ($n\to\infty$);

*then*

1. *there must exist a convergent subsequence* $\{x_{n_{k}}\}$ *of a sequence* $\{x_{n}\}$ *such that* $x_{n_{k}}\to x^{*}$ ($n\to\infty$) *and* $x^{*}\in K$;
2. $\langle T(x^{*}),y-x^{*}\rangle\geq0$, $\forall y\in K$.

#### Proof

In a similar way as in the proof of Theorem A, the conclusion remains. □

### Saddle point problem

Let *B*, *C* be nonempty sets and the function $\phi: B\times C\to R$. The saddle point problem is to find $(x^{*},y^{*})\in B\times C$ such that

$$\phi\bigl(x,y^{*}\bigr)\leq\phi\bigl(x^{*},y^{*}\bigr)\leq\phi\bigl(x^{*},y\bigr),\quad \forall(x,y)\in B\times C. $$

Setting $f((x_{1},x_{2}),(y_{1},y_{2}))=\phi(x_{1},y_{2})-\phi(y_{1},x_{2})$, $\forall (x_{1},x_{2}),(y_{1},y_{2})\in B\times C$ and $A=B\times C$, we have that the saddle point problem is equivalent to (EP).

For each $n=1,2,\ldots$ , let $\phi^{n}: A_{n}=B_{n}\times C_{n}\to R$ be a sequence function and $f^{n}((x_{1},x_{2}), (y_{1},y_{2}))=\phi^{n}(x_{1},y_{2})-\phi ^{n}(y_{1},x_{2})$.

#### Proposition 5.3

*Let* *ϕ*, $\{\phi^{n}\}$, *f*, $\{f^{n}\}$, *A*, $\{A_{n}\}$ *be as in the above note*. *If the assumptions are satisfied*:

1. *For each* $n=1,2,\ldots$ , $\phi^{n}: A_{n}\to R$ *is pseudocontinuous with* $\sup_{(x,y)\in A}|\phi^{n}(x,y)-\phi(x,y)|\to 0$ ($n\to\infty$);
2. *For each* $n=1,2,\ldots$ , $A_{n}$ *is a nonempty subset of* *X* *with* $h(A_{n},A)\to0$ ($n\to\infty$), *where* *A* *is a nonempty subset of* *X*;
3. *For each* $n=1,2,\ldots$ , $(x_{n},y_{n})\in A_{n}$ *satisfies*
  $$\phi^{n}(x_{n},y)-\phi^{n}(x,y_{n})\geq- \epsilon_{n},\quad \forall(x,y)\in A_{n}, $$
  *where* $\epsilon_{n}\geq0$, $\epsilon_{n}\to0$ ($n\to\infty$);

*then*

1. *there must exist a convergent subsequence* $\{ (x_{n_{k}},y_{n_{k}})\}$ *of a sequence* $\{(x_{n},y_{n})\}$ *such that* $(x_{n_{k}},y_{n_{k}})\to(x^{*},y^{*})$ ($n\to\infty$) *and* $(x^{*},y^{*})\in A$;
2. $\phi(x^{*},y)-\phi(x,y^{*})\geq0$, $\forall(x,y)\in A$.

#### Proof

The proof is straightforward and so we omit it. □

## Conclusion

In this paper, we have provided an approximation theorem for equilibrium problems. According to the theorem, there are three similar ways, namely approximate functions as objective functions, similar strategies as practical strategies and approximate calculation methods as practical methods. In Theorem A, we obtain the convergent subsequence of an approximate solutions sequence of approximate problems and the limit belongs to the set of solutions of objective functions. The significance is to illustrate Simon’s bounded rationality theory, and bounded rationality is an approximate way to full rationality. Furthermore, by the methods of set-valued analysis, especially the Fort theorem, we get the generic uniqueness and convergence of solutions of a class of equilibrium problems in the sense of Baire category. Some examples are given to investigate our theoretical results.
